# Management of diabetic foot disease and amputation in the Irish health system: a qualitative study of patients’ attitudes and experiences with health services

**DOI:** 10.1186/s12913-015-0926-9

**Published:** 2015-07-01

**Authors:** Sarah Delea, Claire Buckley, Andrew Hanrahan, Gerald McGreal, Deirdre Desmond, Sheena McHugh

**Affiliations:** Department of Epidemiology and Public Health, University College Cork, Cork, Ireland; Department of General Practice, University College Cork, Cork, Ireland; Mercy University Hospital, Cork, Ireland; Department of Psychology, Maynooth University, Maynooth, Co, Kildare, Ireland

**Keywords:** Diabetes, Foot disease, Lower limb amputation, Health services, Patient experience

## Abstract

**Background:**

Diabetes is an increasingly prevalent chronic illness that places a huge burden on the individual, the health system and society. Patients with active foot disease and lower limb amputations due to diabetes have a significant amount of interaction with the health care services. The purpose of this study was to explore the attitudes and experiences of foot care services in Ireland among people with diabetes and active foot disease or lower limb amputations.

**Methods:**

A purposive sample of individuals who had either active foot disease or a lower limb amputation as a result of diabetes were recruited from the Prosthetic, Orthotic and Limb Absence Rehabilitation (POLAR) Unit of an Irish hospital. One-to-one interviews were conducted in the POLAR unit using a semi-structured topic guide. Thematic analysis was used to identify, analyse and describe patterns within the data.

**Results:**

Ten males participated in the study. Most participants expressed a need for emotional support alongside the medical management of their condition. There were substantial differences between participants with regard to the level of education and information they appeared to have received regarding their illness. There were also variations in levels of service received. Transport and medication costs were considered barriers. Having a medical card, which entitles the holder to free medical care, eased the burden of the patient’s illness. A number of participants attributed some of the problems they faced with services to the health care system as a whole rather than health care professionals.

**Conclusion:**

Results suggest that rehabilitation services should place a strong focus on psychological as well as physical adjustment to active foot disease or lower limb amputations. The delivery of services needs to be standardised to ensure equal access to medical care and supplies among people with or at risk of lower extremity amputations. The wider social circumstances of patients should be taken into consideration by health care professionals to provide effective support while patients adjust to this potentially life changing complication. The patient’s perspective should also be used to inform health service managers and health professionals on ways to improve services.

## Background

Diabetes mellitus is an increasingly prevalent chronic illness that places a massive burden on the individual, the  health care system and society [1, 2]. According to the World Health Organisation (WHO), 347 million people worldwide have diabetes [3]. In 2010, it was estimated that 135,000 adults (8.9 %) aged 45+ years in the Republic of Ireland had diabetes [4]. According to the Organisation for Economic Co-operation and Development (OECD), Ireland has the second highest admission rate for acute diabetic complications with 44 per 100,000 of the population, which is just below the USA’s rate of almost 57 per 100,000 of the population. At the bottom of the scale, the rate for acute diabetic complications in New Zealand is below 10 per 100,000 of the population according to 2007 figures [5].

Foot ulceration and amputations are one of the primary macrovascular complications associated with diabetes. Research shows that more than 80 % of amputations are preceded by foot ulcers [6] with diabetic foot ulcers affecting 15 % of people suffering with diabetes [7]. In Ireland, 50 % of lower limb amputations and 39 % of foot ulcers are a results of diabetes [8]. OECD figures show that Ireland has a relatively low rate of diabetes-related lower-extremity amputations with 10 amputations per 100,000 of the population compared to the USA rate of 36 amputations and Spain’s rate of 26 amputations [5]. Despite this, 2011 witnessed the second highest number of amputation procedures recorded in Ireland at 389, equating to approximately 7–8 amputations each week [9].

There are no national standards that apply to the provision of services to people who have had amputations. Moreover, there are a number of different offices nationwide dealing with the sanctioning and procurement of services such as prosthetics. These differences have potential to impact significantly on the care experiences of patients. While some amputees are eligible for a medical card which entitles them to free medical care including free prosthetics, others who do not have a medical card musts pay the full cost.

Existing research on the experience of people with diabetes and foot disease and/or amputations is focused predominantly on understanding the psychological aspects of life post amputation [10–13]. Other studies focus on prevention and care processes for diabetic foot ulceration and patients’ perceptions and knowledge of foot self-care [14–18]. Important factors discussed in the studies include the value of education in informing and preparing individuals for risks associated with diabetes, and life after amputation including the rehabilitation process [11, 14, 15, 19]; the importance of health professionals in displaying empathy and understanding of patients’ needs; and the significance of considering individual patient circumstances in provision of effective care and treatment [10, 13, 19–21]. There is a lack of research on diabetic patients with active foot disease and/or lower limb amputations and their experience with health services specifically. There is also a lack of knowledge pertaining to diabetes and foot care services in the Irish health system which is characterised by variation in the provision of diabetes services and entitlement to free healthcare. This disparity is highlighted in a 2009 study by McHugh et al. [22] which aimed to investigate the organisation of diabetes care in general practice in Ireland and identify areas for future development. The study found that delivery of diabetes care in Ireland remains largely unstructured. Researchers also found that key challenges to improving diabetes care appeared to extend to the system and organisational level of care delivery. In another study by O’Donnell et al. [23] variations in the structure and provision of diabetes care in Irish hospitals was highlighted where endocrinology-led services have more developed subspecialty structures and access to specialist allied health professionals. It was found that waiting times are longer and discharge rates to primary care are lower than for non-subspecialty led services. The Irish study suggested that hospital-based outpatient care be developed further to ensure that equitable services are provided nationally.

The aim of the study is to gain insight into the attitudes and experiences of foot care services in Ireland among people with diabetes and active foot disease or lower limb amputations. The study also aims to explore what service users feel are the positive and negative aspects of the care they have received and the ways in which services could be improved.

## Methods

### Design and setting

Qualitative data were collected through a series of semi-structured, one-to-one interviews. Participants were recruited from the Prosthetic, Orthotic and Limb Absence Rehabilitation (POLAR) unit in one hospital in the South of Ireland. Staff at the unit see approximately 30 patients per week. Most patients attending the clinic have undergone some form of amputation. The unit provides specialist, interdisciplinary care that is outcome focused for people with limb amputation or congenital limb loss. The programme is a Consultant-led, therapy managed service provided on an outpatient basis for the region of Cork and Kerry. The unit caters for patients across a number of sanctioning offices in Ireland which deal with the procurement and sanctions of prosthetics.

### Recruitment and participants

Patients with diabetes and active foot disease or lower limb amputation were eligible to participate. We employed the National Diabetes Programme definition of active foot disease namely “either an active foot ulcer (defined as full thickness skin break or an active Charcot foot” [24]. Recruitment took place over a six week period from 1^st^August to 10^th^ September 2013. Inclusion criteria included: having active foot disease and/or a lower extremity amputation as a result of diabetes, being 18 years or older, having English as first language and being a current patient of the unit. Patients with foot problems for reasons other than diabetes and those deemed by the clinical staff to be unable to participate in the interview were excluded. Potential participants were identified by the health care team and were approached by the researcher (SD) in the clinic waiting area and invited to participate in the study. Participants were given an information sheet; those who agreed to take part were given a consent form. Of the fourteen potential participants approached, ten agreed to take part, all of whom were male and were public patients- meaning that they received free medical care. One participant had active foot disease on both feet, six had transtibial amputations, and three had transfemoral amputations. Of the ten participants, six had type 2 diabetes and four had type 1 diabetes. All participants were prosthesis wearers. 

### Topic guide

A semi-structured topic guide was drafted by the first author (SD), reviewed by the wider research team and circulated for expert feedback from the health care professionals involved in service delivery (AH, GMG). The topic guide included open-ended questions involving: the patient’s general background (including biographical information and how the patient developed a foot problem etc.), attitudes towards health care professionals/services, education and information received, access to health services, financial costs and how services and experience could be improved. The topic guide was piloted in the POLAR unit using this first version topic guide with a patient who had undergone a below the knee amputation. No substantial revisions were required following the pilot. The guide was used flexibly during the interviews to keep the natural flow of conversation and to allow participants to freely discuss their experiences. The full topic guide can be viewed in Appendix 1.

### Data collection

Interviews took place in a private room at the POLAR unit to ensure confidentiality and privacy. Interviews were audio recorded. Probing was used during interviews to encourage participants to speak more openly about a certain topic. Robson ([25], p283) defines a probe as “a device to get the interviewee to expand on a response when you have a feeling that [they] have more to give”. One of the probes used during interviews included asking participants ‘What exactly do you mean by that?’ and ‘How did you feel about that?’ Another probe involved remaining silent in order to allow the participant to carry on discussing a topic. At the end of each interview, participants were asked if there was anything else they would like to add to ensure that important aspects of the patient’s experience were covered. The average duration of the interviews was 39 minutes. Recordings were transcribed verbatim. Pseudonyms were used throughout the transcripts.

### Data analysis

Inductive thematic analysis was used to identify, analyse and describe patterns within the data. This is a process of coding that does not fit data into a pre-existing model or frame but rather is data driven. This approach provided rich descriptions of the data based on the views of people with diabetes who have foot disease and lower limb amputations- a patient group with unique and personal healthcare experiences. Data were coded and analysed manually, qualitative software was not used in the analysis. The analysis procedure followed the six phases of data analysis outlined by Braun and Clarke [26] which included familiarization with the data, generating initial codes, searching for, naming, defining and reviewing themes and producing a report. The initial codes identified from the interviews were synthesized, merged and refined to generate themes. Broader themes capturing a range of similar patterns within the data were identified as “superordinate themes” while specific categories within these parent superordinate themes were considered “sub themes”.

Data saturation was reached when the themes became more evident, more consistent and more cohesive throughout data analysis, with comprehensive descriptions and examples of each theme being evident [27]. Themes were also identified on a semantic level i.e. within the explicit or surface meanings of the data [26]. This qualitative method of analysis added to the credibility of the study as themes were based on participants’ responses and their wider implications rather than the researchers’ own assumptions [28]. To enhance the rigor of the analysis, an inter-rater process was carried out on a random selection of two of the interview transcripts involving two members of the research team (SD and SMH, the latter analyst was familiar with the aims of the study but was not involved in data collection). This allowed for more in-depth discussion and interpretation of the data with both researchers coming to a consensus on the main themes and relationships within the data.

### Ethics considerations

This study was approved by the Clinical Research Ethics Committee of the Cork Teaching Hospitals and the Ethics Committee of the Hospital in which the interview took place.

## Results

Four superordinate themes were identified: need for supportive interaction with health professionals, different levels of education and information, geographical disparities in access to services and supplies (with sub-themes; financial cost of foot complications and medical card as lifeline) and responsibility of health care system. An additional theme identified during analysis was wider social circumstances complicating and competing with the illness process. The themes and associated sub-themes are outlined below together with excerpts from participants’ accounts. Ellipses are used to represent words missing from quotations.

### Participants

Of the fourteen eligible individuals invited to participate, ten took part in interviews. The ages of the participants ranged from 40 to 72 years (mean = 58 years). One of the participants had active foot disease on both feet and the remaining nine had undergone a lower limb amputation. All of the participants developed these foot complications as a result of having diabetes. Participant characteristics are detailed in Table 1 below.

**Table 1:** Participant characteristics

Pseudonym	Richard	Jack	David	James	Peter	Mark	Liam	John	Louis	Jonathon
Foot disease/amputation status	Active foot disease on both feet	Transtibial	Transtibial	Transtibial	Transfemoral	Transfemoral	Transtibial	Transfemoral	Transtibial	Transtibial
Prosthesis status	Non-applicable	Prosthesis wearer	Prosthesis wearer	Prosthesis wearer	Prosthesis wearer	Prosthesis wearer	Prosthesis wearer	Prosthesis wearer	Prosthesis wearer	Prosthesis wearer
Financial coverage	All public patients									
Age	Range: 40 to 72 years; Mean: 58 years									
Type of diabetes	Four type 1; Six type 2									

### Need for supportive interaction with health professionals

All of the participants generally had a positive attitude towards the health care professionals delivering the services, with few negative incidences reported. Most participants expressed a need for emotional support alongside the medical management of their condition. Their experiences with health care professionals suggested that they valued understanding, empathy, reassurance and communication with health professionals.

*“I’d like to be reassured by the doctor. That we’re going to do the best we can here and God forbid if you do have to amputate it, that’d be the last resort totally.”*(Richard)

For participants like Mark who have been in the health care system for years with multiple illnesses arising from having diabetes, building a good rapport with health care professionals was an important aspect of the care process. However, two participants explained that they were left in *“shock”* by the blunt and direct manner in which their surgeon informed them of their imminent amputation, leaving them *“frightened”* and in *“shock”*.

*“he came into me one morning- into the ward and he says, I’m going to take your leg off…He just frightened the life out of me…He didn’t even introduce himself at all. Shock!”*(Jack)

### Different levels of education and information

There were substantial differences among the participants in regard to the level of education and information they reported to have received regarding their illness. Their level of knowledge ranged from what might be considered an ‘expert patient’ to having a lack of knowledge around their illness. Some participants provided very positive feedback on the level of education and information they received which left them reassured and confident in their own self-care.

*“When I was told I had diabetes, you’re inclined to say, what’s this all about now? But if you’re given diet charts and you’re told all about it and what to have and what not to have. Then you meet the dietitian then and they’re very good. They’re excellent. There was a dietitian- very good all together- and she just told me the consequences.”* (Jonathon)

Receiving information from health care staff was *“vital”* for some participants to teach them to be aware of effective preventative measures but also to make them aware of the services available to them which they might not previously have known about. These included services to support people in the post amputation adjustment process such as disability support groups and home assistance. However, other participants felt less well informed with one admitting:

*“Nothing was explained to me properly…I didn’t get the proper information- what could happen… They had no diabetic center in the hospital- that you could go to someone to talk to and find out more information.”*(Richard)

Although all of the participants reported receiving education and information regarding their illness, some indicated that they were not aware of foot problems pertaining to diabetes until they were treated for a foot ulcer. Many participants also explained that they were aware of what they needed to do in order to look after their feet and minimize the negative consequences of diabetes. However, they admitted that they chose not to follow doctors’ instructions and many took responsibility for the worsening of their condition.

### Geographical disparities in access to services and supplies

Those living rurally experienced a number of difficulties stemming from the lack of services in their local areas, the requirement to travel to designated centres and associated time and travel costs. There was also a significant variation in the waiting times for supplies such as prostheses and wheelchairs. Those living in urban areas also experienced long waiting times for prostheses and wheelchairs in some places. The problems in urban areas related to the lack of wheelchair facilities and sufficient wheelchair parking in hospitals. These problems in the city provided a double burden for those coming into the city from rural areas.

*“In Hospital they’d no proper facilities for a wheelchair in the ward…I wasn’t supposed to be standing but you know to get over to the toilet I had to get out of the wheelchair and walk to the toilet…and Podiatrist was on to me- were you walking? Because she could see the cracks in the foot.”*(Richard)

*“I got the wheelchair before I left the hospital from Hospital 1, yeah. They were very fast with their funding every time…Surgeon told me down in [names hometown] it could take six months to get approved for a prosthesis and I had it within a month.”*(Peter)

Despite this, some participants reported very positive feedback regarding access to services and supplies. Some reported a high level of support post amputation with one participant exclaiming, *“I’m doing more with one leg than I ever did with two legs!”*(Peter).

### Financial cost of foot complications

Various costs accompanied participants’ care including medication, travel and personal expenses.

*“I’m on a good bit of medication. Being up and down then- I have to get a taxi up and down.”*(Louis)

The issue of transport costs was a bigger problem for participants living in rural areas due to distance to health services. One participant explained that even though he was entitled to free public service travel, he was unable to avail of it as the bus service in his local area did not provide wheelchair accessible buses. Because of this, he explained that he was limited to a taxi which could be quite expensive. A number of participants were availing of free private taxi travel which was being covered by the Health Service Executive (HSE). However, as one participant explained, claiming reimbursement was a difficult process.

*“You have to go down to [names town] to fill out forms and see if you can get your money back. That should definitely be made easier.”*(Liam)

### Medical card as lifeline

One support which alleviated the strain of these costs was entitlement to a medical card. All ten participants had a medical card which entitled them to free prostheses and wheelchairs among other medical supplies as well as free GP services and hospital care. A medical card was considered a necessity by all ten participants. One participant described how *“it eases the burden”* associated with his illness explaining:

*“I would probably die without the medical card. Because I wouldn’t be able to afford the care that I need. Not a hope in hell.”*(Peter)

### Responsibilities of the health care system

A number of participants attributed some of the problems they faced with accessing and using services to the health care system as a whole. Participants were keen to stress that staff were not responsible for the weaknesses in the system, as one participant commented *“their hands are tied”*, but rather that problems stemmed from the system itself and the way it was structured. Similarly, another participant expressed empathy for health professionals and explained that they were working within a limited system.

*“They’re doing the best they can with what they’ve got I say…It’s just trying to get the government to put more money in I say and that’d be hard.”*(Mark)

A reduction in level of service as well as lower quality service was also considered by participants to be the responsibility of the health system. One participant explained how his wife was left *“disappointed”* when the allocated hours of her personal carer were reduced. Another participant who was wheelchair bound explained that he was *“angry”* and *“sleepless”* when allocated an apartment himself without the benefit of supportive home care services:

*“The only thing I found that was very, very wrong in my mind was, they never gave me home-help…Just let me fend for myself.”*(Richard)

### Wider social circumstances complicating and competing with illness process

All of the participants had additional personal or psychological stresses which added to the burden of their condition including: changing living arrangements, multiple health issues, family matters and the stress of having to manage medical demands around a personal life.

*“My wife’s grandfather died, a couple of friends committed suicide and stuff like that…so we hadn’t time to go to appointments.”*(Mark)

A key theme which emerged in relation to the competing psychological demands was the importance of social support. Most participants were dependent on a family member, spouse or neighbor to attend appointments. Other participants used a private taxi service. One participants emphasized the importance of receiving practical and emotional support immediately after amputation:

*“if you ask me, after you have your leg off you’d want a lot of help. You’d want someone coming in every day you know? No one came around talking to me.”*(Liam)

The experience described by these participants emphasizes the importance of social support alongside medical management, especially during pre-operation and immediate post-operation phases of the amputation process.

## Discussion

The aim of this study was to explore the attitudes and experiences of people with diabetes and active foot disease or a lower limb amputation and their interaction with healthcare services in Ireland. The positive and negative aspects of the care received as well as potential service improvements were specific foci.

### Importance of emotional support during treatment

Most of the participants expressed a need for emotional support alongside the medical management of their condition. Their experiences suggested that they valued understanding, empathy, reassurance and communication with health professionals. Similar findings were reported in a Swedish study by Sjödahl et al. [19]. Participants expressed a need for direct answers and reassurance from staff. Patients valued staff’s ability to display awareness of and sensitivity to their needs. While some of the participants in this study experienced a lack of empathy from staff when being informed about possible amputation and immediately after amputation, overall participants described most of the health professionals they dealt with as helpful and understanding of their physical and psychological needs.

### Necessity for early education and information

Participants’ level of education and information around their illness varied widely. Some reported a lack of knowledge and understanding about diabetes when first diagnosed. However, many participants explained that, despite receiving information about foot self-care and ways in which they could reduce the ill effects of diabetes, they chose not to follow doctors’ instructions and so took responsibility for the worsening of their condition. Similarly, Johnson et al. [15] suggested that participants found it difficult to accept their diagnosis and tended to disregard preventative advice. Some participants in this study also explained that they were unaware that they had foot disease and in some cases, diabetes, until explicit symptoms of foot ulceration were evident. Similar findings were reported in a recent Iranian study by Aliasgharpour and Nayeri in which it was found that participants were unaware that they had diabetes until explicit symptoms of foot ulceration were evident such as bleeding. They also explained that they did not receive information or training in relation to diabetes and consequential health problems that may result from this disease [16]. However, participants who had multiple chronic diseases reported a high level of education and information regarding diabetes and their foot complication possibly due to their increased interaction with health professionals.

A variety of diabetes structured education programmes have been developed to increase individuals’ application of knowledge of diabetes, self-empower individuals in their diabetes management, provide psychological adjustment to life with diabetes and ultimately improve clinical outcomes [29]. There are special programmes designed for people with type 1 and type 2 diabetes. One of these programmes- X-PERT- is a structured patient education programme that aims to provide people with the confidence, knowledge and skills necessary to self-manage their diabetes. A UK study [30] which assessed the effectiveness of the X-PERT programme on clinical, lifestyle and psychosocial outcomes, demonstrated improved glycaemic control, reduced BMI and waist circumference, reduced requirement for diabetes medication, increased knowledge of diabetes, self-empowerment and self-management skills at 14 months follow-up. In a systematic review by Loveman et al. [31], twenty-four studies were compared on the clinical and cost effectiveness of educational interventions for patients with diabetes with usual care or educational interventions. Results showed education- as part of intensification of treatment- produced improvement in diabetic control in type 1 diabetes with mixed results in type 2 diabetes. Given the mixed results, no clear characterization is possible as to what features of structured patient education may be the most beneficial.

### Variation in provision of services and supplies

One of the main issues this study highlighted was the significant variation in the provision of foot care services and supplies from one region to the next which is a reflection of the current models of service provision operating in Ireland. Some participants reported long waiting times to receive supplies (such as orthoses and wheelchairs) while others did not experience delays in receiving supplies with some even receiving them earlier than expected. Regional differences in access to services have also been reflected in the international literature. For example, in a study conducted in Australia [20], participants experienced difficulties in accessing quality services in regional areas. Long waiting times, difficulties in making appointments and the failure of health care professionals in acknowledging patient self-management knowledge and practice were highlighted.

To reduce the variation in the standard of care in Ireland, the National Diabetes Programme introduced a model of care for the diabetic foot which is intended to provide a structured, systematic and organised approach to addressing the foot care needs of patients with diabetes. It promotes regular foot care and proper screening of risk cases to reduce the incidence of foot ulcers. The integrated model of management and designated care pathway for the diabetic foot starts at the point of diagnosis of diabetes and continues indefinitely. The key feature of the care pathway is the emphasis on foot care provided by an appropriate healthcare professional at a frequency appropriate to the patients’ needs [24]. A number of podiatrists have been recruited to support the introduction of this model of care and address the service deficits [22]. It is hoped that this will make the service more patient focused and efficient. The introduction of a national model of care in Ireland follows the approach of other countries such as the UK where The National Institute for health and Care Excellence (NICE) provides national standardized guidelines for diabetic foot care [32]. The guidelines provide a set of recommendations for primary and secondary care settings in the prevention and management of foot problems. Care of adults and children with type 2 diabetes by these health services is covered in this guide. Topics addressed by the NICE guidelines include: education on prevention and management of foot problems associated with diabetes for both carers and patients; a definition of what constitutes as an increased risk for foot complications; how to identify those at risk; management of the ulcerated foot; primary prevention and how to prevent reoccurrence; and indications for referral to specialists services [32]. However, the Irish health system would be considered different in comparison to the UK in that it is more hospital-centric than the National Health Service (NHS). Hence, the implementation of service change is context specific. Furthermore, a recent systematic review by van Acker et al [33] reported that implementation of guidelines and the setting up of multidisciplinary clinics for holistic management of diabetic foot disorders varies across Europe; specifically, France, Germany, Italy, Spain and the UK. Inconsistencies between treatment guidelines and clinical practice led the authors to conclude that reformation of healthcare services at primary and secondary care levels may be crucial for optimal management of diabetic foot complications.

The importance of these standardized guidelines and improved services for people with diabetes and foot complications has been demonstrated [34]. A longitudinal study in the UK found that improvements in foot care services, including multi-disciplinary team work, lead to significant reductions in total and major amputation rates over an 11-year period. More specifically, results showed a 62 % decrease in the incidence of major amputations from 7.4 to 2.8 per 100,000 of the general population. Also, over the 11-year period, total amputations decreased by 70 % from 53.2 to 16.0 per 10,000 of people with diabetes, with major amputations falling by 82 % from 36.4 to 6.7 per 10,000 of people with diabetes [34]. This study highlights the importance of monitoring and evaluating changes in health service delivery at a population level. Also, a systematic review by Buckley et al. [35] concluded that there is insufficient evidence to determine whether contact with a podiatrist has an effect on the risk of lower extremity amputation in people with diabetes. However, some of studies included in the review suggest that contact with a podiatrist had a positive effect on shorter-term outcomes, including patient knowledge of foot care and ulcer recurrence. The authors advised that looking at the effects of a multidisciplinary team as oppose to one service in isolation may provide a more accurate reflection on how patients with diabetes are managed [35]. These findings are useful in informing the health care system on approaches to monitoring effective foot care services.

### Responsibility of health care system

Many of the participants attributed problems they faced in their treatment to the health care system as a whole. These issues included delays in accessing services and supplies and a shortage of health care staff. In a study by Feinglass et al. [36] conducted in the United States of America, over half of patients interviewed blamed the health care system for delays in treatment. In the current study, such attributions illustrate an evident tension in the health system in the context of reduced funding, increased demand for services and a moratorium on recruitment of health care professionals to diabetic foot care services across Ireland.

### Taking into consideration wider social circumstances

An important finding in this study was the affect that wider social circumstances had on participants’ illness experience and treatment. All ten of the participants described multiple factors in their personal lives which added to the burden of their illness and sometimes acted as a barrier to accessing services and appointments. It has been recommended that clinicians consider patients feelings towards personal circumstances and how these impact their unique quality of life [21]. Taking these factors into consideration is important in order for health professionals to gain a deeper understanding of the effects that lower extremity ulcers have on people with diabetes while also enhancing their ability to provide sufficient support during the illness process.

### Strengths and limitations

In light of the lack of research on the health service experiences of people with diabetes and active foot disease and/or lower limb amputations, this paper addresses the dearth of research on the experiences of people in this population group and their interaction with the health services. This is an extremely important topic given the increases in the number of patients with diabetes and foot complications and the frequency with which they are in contact with health care professionals across different settings.

The flexibility afforded by the semi-structured interview process together with the application of thematic analysis might be considered strengths of the study as they allowed for the description and exploration of novel areas of concern reported by the participants. For example, in one of the early interviews in this study, a participant was asked if there was anything in particular that added to the stress of his condition. On replying to this question, the participant mentioned the limited parking available at the hospital. This factor was then mentioned by the researcher in further interviews and it was found to be a common concern among participants. This factor may not have been brought to the researcher’s attention had a “pre-determined question” been asked ([37], p293). Furthermore, an inter-rater process was carried out with two of the interview transcripts involving another researcher. This allowed for more in-depth discussion and interpretations of the data with both researchers coming to a consensus on important themes.

Although every effort was made to enhance to quality of this study, it is not without limitations. Firstly, this study’s population sample was limited to Caucasian males. Therefore, female perspectives and those of different ethnic backgrounds were not accounted for. This was due to the limited data collection period, the convenience sample of those attending the clinic over summer and because patients of the POLAR Unit are predominantly male. Secondly, only one of the participants had active foot disease alone while the remaining nine had undergone a lower extremity amputation. Therefore, the majority of the data is taken from the experience of people who had undergone the amputation process. However, these participants were also asked about the health care they received prior to operation.

## Conclusion

The distress and anxiety that the participants in this study felt was reduced by health professionals who were sympathetic and reassuring, providing participants with emotional support alongside medical care. Early education is vital in informing people with diabetes about the foot risks they face without careful preventive measures and self- management. With significant variation in levels of service across different regions in the country including variations in waiting times for prostheses, a standardized provision of services and supplies is needed to ensure every individual with or at risk of an amputation receives equal access to and delivery of urgently needed care and medical supplies.

Most of the problems described by participants were a result of issues with the structure and coordination of the national health care system as a whole. Patients’ wider social circumstances must be taken into consideration in order for health professionals to gain a deeper understanding of the effects that lower extremity ulcers and amputations have on people with diabetes while also enhancing their ability to provide sufficient support during the illness process.

## Appendix 1: Topic guide

### General background

Name/D.O.B./Country of origin/ When did you first notice a problem with your feet? How did you first start coming to the clinic?

### Attitudes towards health care professionals/providers

Who was the first health professional you met when you got a foot ulcer/before your amputation? (Doctor, nurse, GP) What has been your overall experience with foot care services to date? What has been your relationship like with your doctor? Do you feel you were actively involved in the management of your feet?

### Foot self-care

Tell me about the things you do yourself to look after your feet? Is there any one at home who helps you? How do they help you?

### Education/information received

What kind of information were you given on the treatment of your feet and foot self-care? How were the risks associated with feet in people with diabetes explained to you? Were you told about these risks before your foot ulcer/amputation? How did you feel when your foot problem and treatment was explained to you?

### Access to services

How has your experience been with access to foot care services? Were there for example, delays or long waiting times? Were you waiting long to receive a wheelchair/prosthesis?

### Financial costs

Are you a public or private patient? How has this affected you? Has it added to or taken away from the stress of your condition?

### How could services/experience be improved?

Are there certain aspects about the services you are receiving for your feet that you feel could be improved? If you could change one aspect of the health services you receive/received, what would it be?

## Abbreviations

POLAR Unit: Prosthetic, Orthotic and Limb Absence Rehabilitation Unit
OECD: Organisation for Economic Co-operation and Development
HSE: Health Service Executive
